# Wireless communications sensing and security above 100 GHz

**DOI:** 10.1038/s41467-023-36621-x

**Published:** 2023-02-15

**Authors:** Josep M. Jornet, Edward W. Knightly, Daniel M. Mittleman

**Affiliations:** 1 Department of Electrical and Computer Engineering, Institute for the Wireless Internet of Things, Northeastern University, 360 Huntington Avenue, Boston, MA 02115 USA; 2 Department of Electrical & Computer Engineering, Rice University, 6100 Main St., Houston, TX 77005 USA; 3 School of Engineering, Brown University, 184 Hope St., Providence, RI 02912 USA

**Keywords:** Terahertz optics, Fibre optics and optical communications

## Abstract

The field of sub-terahertz wireless communications is advancing rapidly, with major research efforts ramping up around the globe. To address some of the significant hurdles associated with exploiting these high frequencies for broadband and secure networking, systems will require extensive new capabilities for sensing their environment and manipulating their broadcasts. Based on these requirements, a vision for future wireless systems is beginning to emerge. In this Perspective article, we discuss some of the prominent challenges and possible solutions which are at the forefront of current research, and which will contribute to the architecture of wireless platforms beyond 5G.

## Abstract (web summary)

The field of terahertz wireless communication is growing rapidly. Here the authors discuss the challenges for the architectures of wireless platforms above 100 GHz and their potential applications.

## Introduction

Over the last two decades, wireless communications exploiting radio-frequency waves have become a ubiquitous feature of modern life. With each subsequent advance in technology has come countless new tools and capabilities, transforming the way we live. Now, as the rollout of 5G systems continues, researchers are considering the design of subsequent generations of networks, as well as visions for future implementations of Wi-Fi, Bluetooth, and other short-range wireless systems. It is worth noting that most previous wireless platforms, from the days of Marconi, have been confined to operate in the frequency range below a few gigahertz. Yet, rapid growth in demand for wireless services has changed the game; we are now forced to consider using higher frequencies, in order to find the bandwidth needed to support continued exponential growth in wireless traffic. One of the novel features of modern Wi-Fi and 5G variants such as IEEE 802.11ay^1^ involves their ability to access higher frequencies in the millimeter-wave range, above 10 GHz. As these systems mature, it is therefore natural that research interests have now begun to turn to even higher frequencies. Like the mmWave Wi-Fi and 5G bands, the use of these higher frequencies is motivated in large part by the desire for access to larger bandwidth, and the associated higher data rates. Indeed, although the maximum data rate that can be supported within the 5G standard exceeds 7 gigabits per second (Gbps), more than an order of magnitude larger than the fastest 4G data rate, the huge (and ongoing) growth in demand for wireless access has made it clear that even higher rates will be needed in the future^2^.

For this reason, the cutting edge of wireless research lies at frequencies above 100 GHz^3^, often referred to as the “terahertz (THz) range”. Most of this research is focused on a few specific broad spectral bands, including the waveguide D band (110−170 GHz) which has been previously employed for television broadcasts during the Beijing Olympics^4^, and the higher frequency bands defined by the recent IEEE 802.15.3d standards document (252−322 GHz)^5^. These frequencies are well beyond even the highest millimeter-wave bands included in today’s Wi-Fi and 5G standards.

Opening this relatively unexplored realm of the electromagnetic spectrum will involve a host of challenging new research problems. In this Perspective article, we discuss some of the interesting issues facing researchers in the race to develop ultra-high-frequency wireless systems. Many of these challenges are associated with aspects of the physics of the interaction of these high-frequency waves with the world. Above 100 GHz, system designers will need to consider some physical regimes that have not previously been relevant for legacy wireless systems, or even in some cases for the mmWave bands of 5G. We first consider a few of the more prominent issues associated with these new operating regimes. We note that near-infrared or visible light optical communication systems, operating at even higher frequencies, are also of significant research interest, but are beyond the scope of this article.

### Some challenges

One aspect of this discussion can be understood from the Friis equation, which describes the power received by an antenna *P*_RX_ in a line-of-sight point-to-point wireless link. Expressed in dB, this relation is:

1 $${P}_{{{{{{\rm{RX}}}}}}}={P}_{{{{{{\rm{TX}}}}}}}+{G}_{{{{{{\rm{T}}}}}}}+{G}_{{{{{{\rm{R}}}}}}}{-}20\,{\log }_{10}(4{{{{{\rm{\pi }}}}}}D/\lambda )$$

**Fig. 1:**
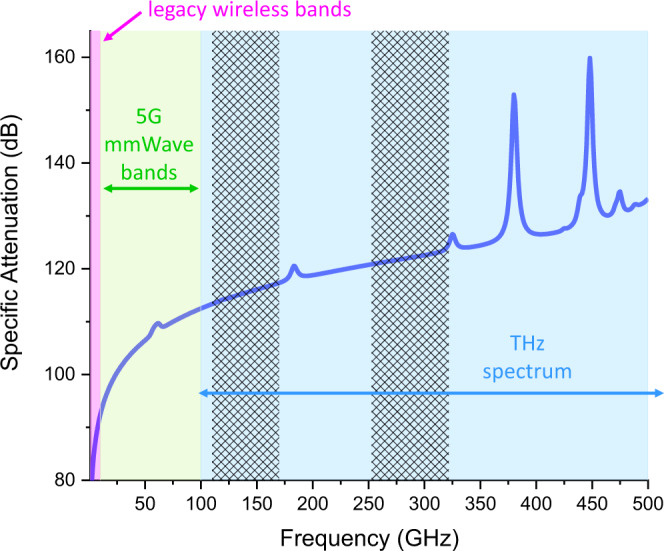
Atmosphere and FSPL. The attenuation of a propagating radio wave due to both free-space path loss and atmospheric absorption, for an assumed propagation distance of 100 m, at a temperature of 15 °C and relative humidity of 59%, using a standard atmospheric model (see [10, 11]). The shaded areas indicate the range of frequencies corresponding to legacy wireless systems, the 5G millimeter-wave range, and the THz spectrum. The hatched areas are the two bands of significant interest for communications mentioned in the text.

Here, *P*_TX_ is the power generated by the transmitter, *G*_T_ and *G*_R_ are the transmitter and receiver antenna gains respectively (in dBi), and the last term, the free-space path loss (FSPL), describes the decrease in power per unit area of an expanding electromagnetic wavefront in terms of the propagation distance *D* and the wavelength λ^6^. This term becomes dominant at high frequencies. When considering an increase in the frequency by a factor of 100 (for example, from the typical 4G cellular frequency of 2.8 GHz to 280 GHz, a frequency in the 802.15.3d standard), the FSPL increases by 40 dB (see Fig. 1). Because the FSPL is smaller at lower frequencies, high-gain antennas are not always required; it is possible to operate a wireless link in which the transmitter broadcasts to a wide range of angles. For example, typical cellular antennas often span a 120° broadcast sector. At higher frequencies, the increasing FSPL can be offset with high-gain antennas, which concentrate the radiated power into a smaller angular range. Above 100 GHz, these broadcasts begin to act more like beams, propagating in a well-defined direction with low divergence^7^. There are of course many possible options for high-gain antennas, but translating these to the THz range is not always trivial, due to (among other things) the requirement of broadband operation. For example, phased array antennas are a well-established technology at lower frequencies, employing tuned phase shifters for each of the antenna elements in an array to implement beam steering or wavefront shaping. This approach, also being used in 5G systems, becomes more challenging as we design systems with larger fractional bandwidths. Phase shifters commonly operate at a fixed wavelength or frequency. When injecting a broadband signal to a phase shifter, the different frequency components experience different phases, resulting in beam squinting. Instead, a true-time-delay operation, in which all the signal frequency components experience the same phase delay, may be required in place of a simple phase shifter for individual elements of an antenna array^8^. The design of active efficient high-gain antennas with suitable form factors and efficiency remains an important research challenge.

A second important distinction between low and high-frequency propagation involves atmospheric attenuation (which has been neglected in Eq. (1) above). This loss also increases with frequency, in the form of several spectrally narrow absorption peaks riding on top of a smoothly increasing continuum absorption. In terrestrial systems, all of the important absorption lines above 120 GHz are due to rotational and ro-vibrational excitations in gas-phase water molecules^9,10^, some of which are strong enough to inhibit long-range propagation for frequencies near their line centers. These discrete absorption lines, therefore, serve to break the spectrum up into a series of broad bands which are well suited for transmission over longer distances, in which the relatively small continuum background (due to water dimers and other species) is the dominant contribution to atmospheric loss^11^. In fact, these absorption resonances need not always be considered a hindrance; with careful frequency tuning, they can be exploited for enhanced wireless security^12^. Despite some older conventional wisdom, the atmosphere is not opaque to radiation in the 100–1000 GHz range; if the H_2_O lines are avoided, point-to-point links in the km range are certainly feasible^4,13–15^. Inclement weather also contributes additional loss;^16,17^ however, these may be tolerable under certain conditions, and indeed terahertz beams appear to be more robust against atmospheric scintillation and certain weather conditions (e.g., fog) than, for example, free-space optical signals in the near-infrared^18^.

**Fig. 2:**
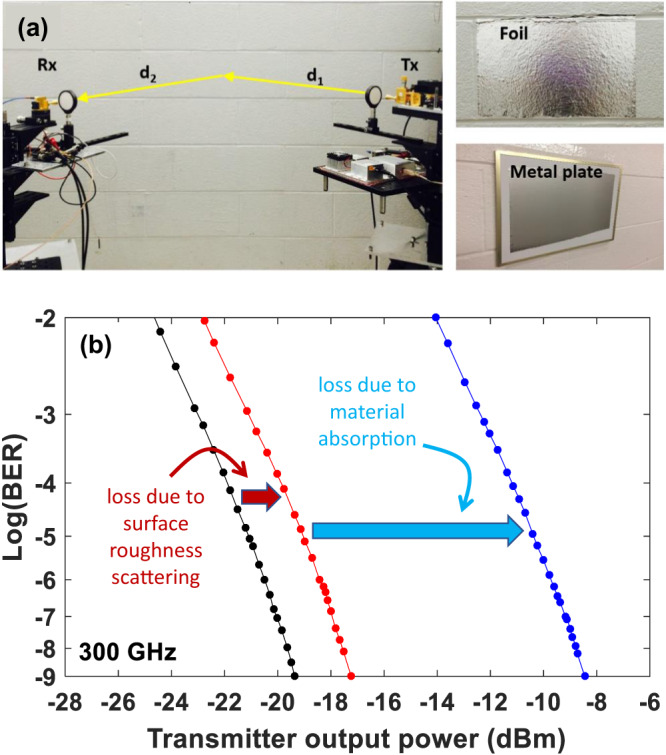
Links employing specular reflection. Measured bit error rate (BER) for a 2-m link which incorporates a specular reflection from a cinderblock wall, as shown in the top left photo. The effects of absorption and scattering are separated by measuring the link on the bare wall (blue points), the same wall with a conformal metal foil coating that eliminates penetration into the cinderblock (red points), and a flat metal plate which eliminates both absorption and scattering (black points). The photo images in (**a**) depict the three situations corresponding to the measurements in (**b**). Reprinted from Ma, J., Shrestha, R., Moeller, L. & Mittleman, D. M.; Channel performance of indoor and outdoor terahertz wireless links. APL Photon. 3, 051601 (2018)., with the permission of AIP Publishing.

A third issue of note is of the roles of scattering from surfaces and of material absorption. When considering interactions with surfaces, the characteristics of the scattered field are determined by the roughness of the surface, in comparison with the wavelength of the radiation, as well as the extent to which the surface absorbs (rather than scatters) the incoming radiation. A smooth (compared to lambda) surface reflects like a mirror; a rough surface produces a diffuse (not strongly directional) scattered wave. In a typical indoor environment, for instance, conventional wireless systems operate at frequencies where absorption is low in many materials, and where many surfaces are smooth compared to the (longer) wavelength. So, it is generally assumed that there can be many multiply-scattered paths between the transmitter and receiver, producing a rich scattering environment in which the field at any location is a stochastic superposition of many different wavelets. In contrast, the wireless channel at THz frequencies is quite different^19^. Typically a propagating wave experience much higher attenuation when interacting with most surfaces, due to absorption losses in the materials^20^, and commonly encountered surfaces can either be smooth or rough, in comparison with the (much smaller) wavelength (see Fig. 2). As a result, both indoor^21^ and outdoor^22^ environments are typically much more sparse, with fewer paths connecting the transmitter to receiver. Because many surfaces are smooth enough to act like mirrors, scattering in a specular direction (i.e., angle of reflection = angle of incidence) can often be dominant. Researchers have therefore been able to exploit ray tracing as an accurate means for predicting and understanding signal paths in THz propagation simulations^21,23^. In addition, due to the opacity of many objects including people, issues such as blockage of the direct line-of-sight (LOS) path can pose challenges for maintaining connectivity, as would be the case with a laser-based free-space optical link. However, due to the millimeter-scale wavelength, steering around such blockage events by exploiting a specular reflection from a surface in the environment is more feasible at THz frequencies^24^. Even non-specular reflections (diffuse scattering from rough surface^25^) can be employed to maintain a link, although obviously with a lower signal-to-noise ratio^26^ and added dispersion^27^. The shift from omnidirectional broadcasts with rich scattering to directional beams with sparse paths also has important implications for the security of such communication channels, rendering eavesdropping more challenging. Yet, vulnerabilities due to scattering still remain^28–30^, and must be considered in system design.

### Defining wavefronts and waveforms

These various novel features of THz waves force us to rethink common practices in wireless communication systems and, at the same time, open the door to new strategies not available in traditional wireless networks in microwave and even millimeter-wave bands.

On the one hand, relating to the spatial behavior of terahertz radiation, the requirement for high-gain directional antennas strongly suggests the use of radiating structures that are much larger than the wavelength. By recalling that the far field of an antenna occurs for distances greater than 2 × D^2^/λ, where D is the antenna’s largest dimension, it is likely that many wireless systems at terahertz frequencies will operate in the near field. For example, a 10 cm antenna, such as a dish antenna or an antenna array, at 130 GHz has a far-field distance of 8.6 m and the same antenna size at 300 GHz has a far-field distance of 20 m, larger than many indoor environments in which a THz LAN could be employed. This is a major distinction from lower frequency wireless systems, which generally operate exclusively in the far field.

This result has multiple consequences. First, wireless propagation, channel, and multi-user interference models, which have been derived under the assumption of far-field operation^6^, cannot simply be repurposed for higher frequency systems. Indeed, many models for terahertz communications continue to neglect to capture near-field effects^31^. Second, most algorithms behind the control of smart directional antenna systems, including beamforming and beam-steering, have also generally been developed under the far-field assumption^32^. For many possible antennas, including large radiating structures such as the increasingly popular intelligent surfaces^33^, this is not the case even at lower frequencies.

To overcome this latter challenge, there are several recent works^34,35^ that explore beam focusing as a way to achieve beamforming-like capabilities but in the near field. In beam focusing, the weights or phases at different antenna elements are set to emulate that of a dielectric lens. While this is a valid solution for static scenarios, tracking and constantly changing the point on which the signal needs to be focused results in a significant overhead in terms of signaling the channel state information.

Going beyond beam focusing, if we are ready to abandon common practices and assumptions such as that the generated signal can be approximated as a plane wave or a Gaussian directional beam, operating in the near field opens the door to a host of new possibilities in wavefront engineering (whereby wavefronts we refer to the spatial intensity and phase profiles of the signals being transmitted). Although many of these ideas have been considered for some time, for instance in the optics community, it is only with the advent of directional links that they may reach their full potential in wireless systems. For example, at lower frequencies where received signals can often contain rich multi-path components, it can be challenging to exploit polarization diversity to double channel capacity. In contrast, such strategies are likely to be far more effective with a line-of-sight directional link^36^.

**Fig. 3:**
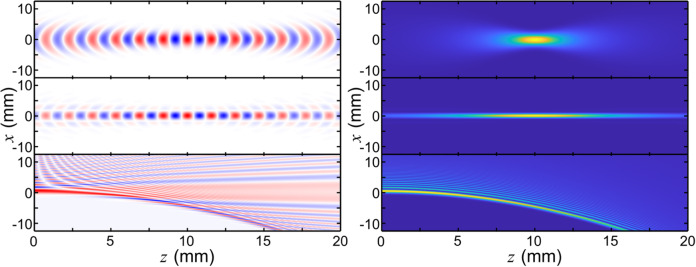
Engineered near-field radiation patterns. Calculated electric field (left) and intensity (right) patterns for three engineered near-field radiation patterns: a focused Gaussian beam (top), a Bessel beam (middle), and an accelerating Airy beam (bottom).

Other important examples may arise from considerations of more exotic wavefronts which can be prepared in the near field of an emitting aperture. For instance, by adopting Bessel beams, i.e., beams whose intensity profile in space can be described by a Bessel function of a certain order^37^, a beam can focus (in the near field) not at a point but along a line. This can drastically simplify the operational requirements in mobile networks. Moreover, Bessel beams exhibit a self-healing property, i.e., even when partially blocked by an obstacle, they can recreate the original intensity and phase profile at a distance. Similarly, the use of accelerating beams such as Airy beams, which can be programmed at the origin to bend after a given number of wavelengths^38^, can also be utilized to overcome or minimize the impact of obstacles, a major problem for practical mobile terahertz communications and sensing systems. Figure 3 shows computed cross-sections of a few of these options, illustrating the dramatically different behavior that can be obtained in the near field of a transmitting aperture.

Further, all these beams can also be engineered to carry orbital angular momentum (OAM). Different OAM mode orders are orthogonal, enabling the multiplexing of streams at the same frequency, at the same time, and in the same direction^39^. As discussed in the literature^40^, OAM multiplexing can be seen as a particular case of multiple input multiple output (MIMO) communications, but one in which channels are orthogonal from the start (and not because of how multi-path propagation affects them). Moreover, while these wavefronts can be generated using static phase masks (such as axicons for Bessel beams or spiral phase plates for different OAM modes), the same can be achieved by the utilization of dense antenna arrays^41,42^, which (unlike phase masks) could also in principle be dynamically reconfigurable^43,44^. We note that the security vulnerabilities associated with using such unusual wavefronts could be quite different from those associated with conventional side-lobe eavesdropping or jamming attacks^45^, and could offer new opportunities for enhancing link security^46^.

**Fig. 4:**
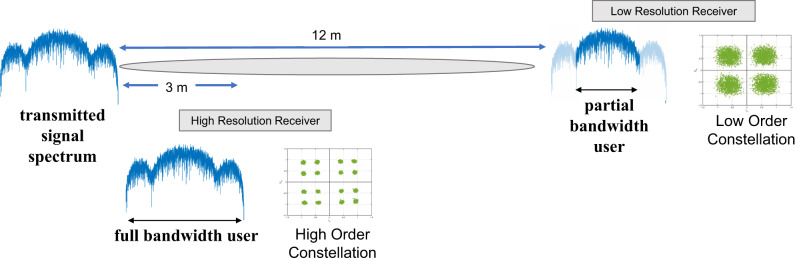
Hierarchical bandwidth modulation. Leveraging the spectral filtering effect of atmospheric water vapor absorption resonances to implement hierarchical bandwidth modulation, in which nearby users can access the full bandwidth of the transmitted signal, while more distant users, whose channel bandwidth is narrower, only employ the smaller range at the center of the spectrum.

On the other hand, relating to the frequency behavior of terahertz radiation, there is a need for waveforms, the temporal variations of the transmitted signals, that can overcome various challenges, including those introduced by frequency-dependent molecular absorption in the channel (see Fig. 1) and by increasingly prominent hardware imperfections (e.g., nonlinearities in broadband frequency up- and down-converting systems). As of today, there is no answer to the question of what waveform will be used for 6G terahertz systems. While the common solutions at lower frequencies, including orthogonal frequency division modulation/multiplexing (OFDM), single-carrier OFDM also known as DFT spread OFDM, or the recently proposed orthogonal time-frequency-spatial modulation (OTFS)^47^ could be adapted to terahertz frequencies, there are also other options, including waveforms unique to the terahertz band that enable applications not available at lower frequencies. For example, very short pulses, just a few hundreds of femtoseconds long, as in terahertz time-domain spectroscopy (THz-TDS) platforms^48^ or BiCMOS impulse radiators^49^, can be utilized to implement low-complexity non-coherent modulation which is able to support a large number of users transmitting at very large data rates over a short range, provided that proper equalization techniques are implemented to compensate for the effects of multi-path propagation^2,50^. This is particularly useful when the encoding purposely biases the transmission of zeros over the transmission of ones to overcome the impact of noise and interference. At the same time, for longer communication distances, the broadening of the molecular absorption lines results in narrower communication bandwidths at longer distances. This effect can be exploited to use the channel as a filter and help to separate simultaneous data streams at the same frequency for users in the same direction but at different distances (see Fig. 4)^51^. Moreover, if spectral efficiency and peak data rates are not the drivers, there are other ways to exploit the available bandwidth above 100 GHz, for example in the form of secure communication and spectrum sharing techniques based on ultra-broadband spread spectrum^52^.

Ultimately, we envision that the spatial and spectral aspects of terahertz signals should not be considered separately, but instead, the spatial wavefront and temporal waveform should be jointly designed to optimize the performance of systems and unleash this spectrum. For example, as discussed above, different wavefronts are commonly generated with different types of phase masks which can be, in some cases, intrinsically narrowband. However, when trying to transmit ultra-broadband signals through such structures, the resulting wavefronts are far from ideal. To prevent this, frequency-selective pre-distortion of the waveforms being transmitted can be employed to ensure that the desired wavefront is produced over the entire bandwidth. This becomes even more important when producing complex wavefronts using arrays that approximate the lens response with a discrete (rather than continuous) pattern.

### Components for the physical layer

Of course, the impact of the unique properties of terahertz radiation does not end with propagation, wavefronts, and waveforms. It will also influence the redesign of common devices in traditional systems for operation at higher frequencies and will open the door to novel hardware solutions that are not practical or even possible at lower frequency bands.

As one example, the small wavelength of terahertz waves leads to small fundamental resonant antennas (e.g., dipole, slots, or patches). When used individually, these antennas exhibit low effective areas resulting in the very high spreading losses discussed above. However, it is this small size of radiating structures that allows us to integrate very large numbers of antennas in a very small footprint. For example, in a 10 cm × 10 cm footprint, one could in principle integrate 200 × 200 (40,000!) dipole antennas at 300 GHz spaced λ/2 apart. The fabrication of such large on-chip arrays is a significant challenge, but rapid progress is being made^43,44^.

While such antennas or radiating elements can be envisioned, there are other components besides the antennas that would need to be integrated into the chip (potentially through 3D stacking), depending on the application that is needed. For example, a true-time delay controller per element would be needed to engineer the aforementioned broadband wavefronts. Moreover, if the goal were to develop transmitting or receiving antenna arrays that can support MIMO communications, each antenna would require a complete RF chain (i.e., a local oscillator, mixer, filter, amplifier, and data converter). Integrating such arrays is a major bottleneck with today’s electronic and photonic transceiver technologies due to their size, as well as packaging and thermal constraints. Arrays with element spacing greater than λ/2 produce far-field radiation patterns with grating lobes, which could be leveraged for multi-beam systems, but are otherwise not desirable. Instead, antenna array architectures aimed at minimizing the number of RF chains while minimally impacting the array capabilities have been proposed, such as the array-of-sub-arrays architecture^47^, in which separate RF chains drive separate subsets of fixed or only phase-controlled antenna elements^53^. Other solutions could be the adoption of signal processing techniques for sparse antenna arrays, which so far have generally been used only in the context of imaging^54,55^.

There are also a number of new technologies that only become available when operating at terahertz frequencies (or above). For instance, researchers have proposed the use of graphene to build plasmonic transceivers and antennas that intrinsically operate in the terahertz band^56^. Graphene, which supports the propagation of surface plasmon polariton (SPP) waves at terahertz frequencies and at room temperature, can be used (1) as a two-dimensional electron gas where plasma waves oscillations at terahertz frequencies occur^57^, (2) as a plasmonic waveguide where the properties of SPP waves can be electrically tuned^58^, and (3) as the active element of a nano-patch antenna, able to convert SPP waves into free-space electromagnetic waves^59^, all with devices that are significantly smaller than the free-space wavelength. While being sub-wavelength in size leads to low radiation efficiencies, this can be compensated through dense integration of the elements. Moreover, the sub-wavelength nature of each radiator also leads to negligible mutual coupling as long as elements are placed more than a plasmonic wavelength apart. From a signal processing perspective, being able to sample space with a resolution higher than λ/2 leads to both oversampling gain and the ability to engineer wavefronts (such as those noted above) with much higher accuracy than traditional λ/2-spaced arrays could ever support^60^.

The shift to higher frequencies also offers fascinating opportunities to engineer devices with advanced functionality, which are either impossible or impractical at lower frequencies. For example, recent research has focused on leaky-wave antennas, based on guided wave devices which incorporate a mechanism to permit some fraction of the guided wave to ‘leak’ out into free space. This leaked signal manifests a strong coupling between the frequency of the radiation and the direction in which it propagates. Leaky-wave antennas are neither new nor exclusive to the terahertz range^61^. However, the wavelength scale, and spectral bandwidth, of signals at these high frequencies means that such devices can operate in a unique regime of form factor and functionality, such that it is now plausible to consider new roles for these components in wireless systems. Leaky-wave components can be valuable for multi-frequency signal distribution (i.e., multiplexing)^62^ and for sensing tasks such as the radar-like location of objects within a broadcast sector^63^. If both transmitter and receiver are equipped with a leaky-wave antenna, they can together provide a fast and efficient method for simultaneously determining both the angular location of a mobile receiver and its angular rotation relative to the transmitter^64^. Building on this approach, recent work has demonstrated arrays with true-time-delay elements to accomplish similar localization tasks^65^. One could even envision creating arrays of leaky-wave devices for enhanced wavefront control. This is another idea which has previously been considered at lower frequencies^66^, but which could find new possibilities in a different frequency regime.

**Fig. 5:**
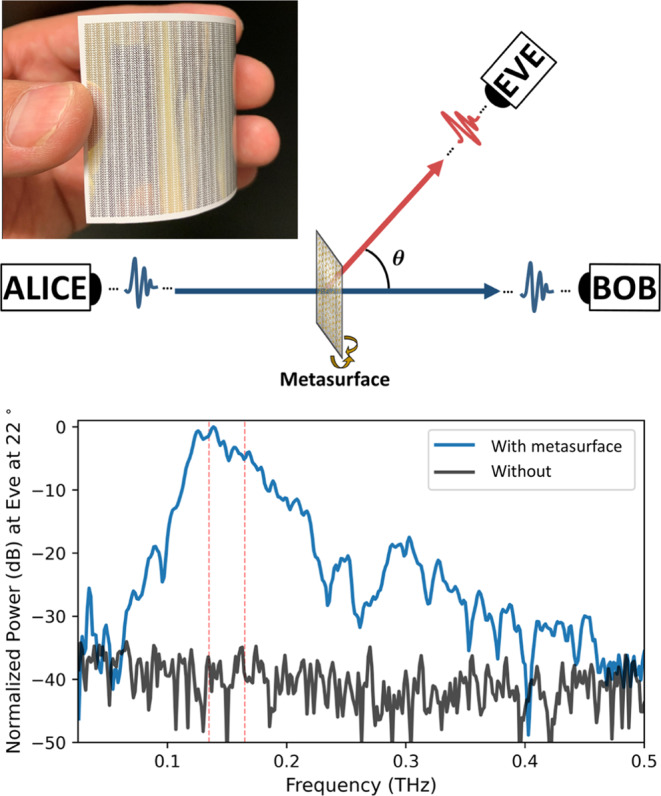
Metasurface-in-the-middle attack. A clever eavesdropper (Eve) can insert an engineered reflector, such as a flexible metasurface (photo) into the line-of-sight path between the transmitter (Alice) and the intended receiver (Bob), in order to direct a portion of the spectrum towards the eavesdropper (upper schematic). This low-profile attack would be difficult for Alice and Bob to detect, but would direct a significant signal toward Eve (lower panel).  Adapted from Zhambyl Shaikhanov, Fahid Hassan, Hichem Guerboukha, Daniel Mittleman, and Edward Knightly. 2022. Metasurface-in-the-middle attack: From Theory to Experiment. In Proceedings of the 15th ACM Conference on Security and Privacy in Wireless and Mobile Networks (WiSec '22).© Association for Computing Machinery, New York, NY, USA, 257–267. 10.1145/3507657.3528549.

The challenging propagation of terahertz waves and, in particular, the issue of blockage, motivates the consideration of strategies to improve reliability. As noted above (see Fig. 2), non-line-of-sight paths are available, even at these high frequencies, although they are sparse relative to what is typically encountered at lower frequencies. One interesting approach relies on the development of devices that can help us engineer not only the transmitter and receiver but also the propagation environment (i.e., the channel). This idea has inspired a great deal of research in the general area of intelligent reflecting surfaces, which could be distributed throughout an indoor network to facilitate signal distribution and overcome transient blockage events. As with other devices discussed here, an intelligent reflecting surface (IRS), such as those based on programmable reflectarrays, has been considered previously at lower frequencies^67^. However, at frequencies above 100 GHz and with current applications in mind, the benefits that such structures bring to wireless systems may now prove too valuable to ignore. Today, there are numerous different technologies under consideration as the basis for IRS devices. For example, smart surfaces have been proposed which replace conventional switching elements employed at lower frequencies, such as varactor diodes, with graphene patches^56^. Dense reflectarrays with integrated switching elements have been designed and implemented in silicon CMOS^43,44^ and in III-V semiconductor platforms using high-electron-mobility transistor (HEMT) structures^68^. We have also recently shown that array devices, in the hands of a clever adversary, can also open up interesting new security vulnerabilities (see Fig. 5)^69^. It is too early to tell how this interesting approach to engineering the broadcast environment will ultimately be achieved, but it is quite clear that any of these possible solutions would drastically change the way that networks are designed and operated.

### Implications for the control plane

With the above considerations in mind, it becomes clear that networks of the future, which have the capability to exploit THz frequency bands, will operate quite differently from networks of today. One obvious example is that a transmitter, employing a narrow pencil-like beam, will need to know where to point it. This and other examples suggest that networks will require joint communications and sensing capabilities, and moreover that new approaches will be required for managing these capabilities in order to ensure high quality of service and efficient use of system resources.

As a first step, we note that radio sensing can have two purposes: The first purpose is to identify clients, devices, and objects in the environment, e.g., for presence detection and analysis of environmental objects and their mobility to optimize the signal-to-noise of wireless links^70^. The second purpose builds on the first and targets to understand the RF environment for network optimization, e.g., to localize uncontrolled sources of interference in order to avoid or null them. Today’s RF sensing applications are quite impressive and include monitoring people in a room behind a wall^71^ and monitoring individual heart rate^72^. Unfortunately, today’s RF methods have two fundamental limits. First, their inputs are the gains and phases of the channel matrix *H* and they subsequently rely on the dimensionality of *H* for resolution. Thus, to improve resolution further, array sizes would need to approach a massive MIMO scale, thus incurring the corresponding issues of size, cost, power consumption, and computational requirements. Second, because these methods were designed to operate below 6 GHz, their wavelength is centimeter to decimeter scale, limiting resolution correspondingly^73^.

**Fig. 6:**
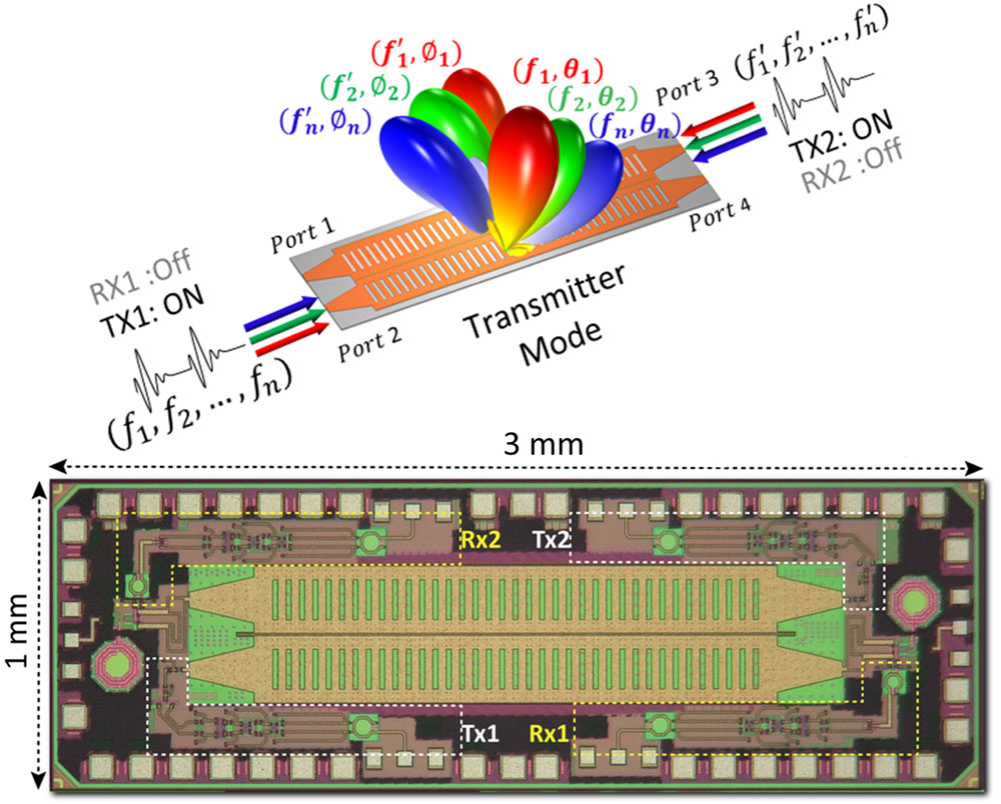
CMOS leaky-wave emitter. An integrated circuit, fabricated in silicon CMOS, which realizes a leaky-wave antenna for single-shot localization of multiple users in a broadcast sector via broadband excitation of the angularly dispersive aperture. Figure adapted from H. Saeidi, S. Venkatesh, X. Lu and K. Sengupta, "THz Prism: One-Shot Simultaneous Localization of Multiple Wireless Nodes With Leaky-Wave THz Antennas and Transceivers in CMOS," in IEEE Journal of Solid-State Circuits, vol. 56, no. 12, pp. 3840-3854, Dec. 2021, 10.1109/JSSC.2021.3115407. with permission of the authors under a Creative Commons license: https://creativecommons.org/licenses/by/4.0.

As noted above, the use of THz frequencies opens up a number of new possibilities for joint sensing and communications, with important implications for the functioning of the control plane of the network, which is responsible for functions such as beam alignment and spectrum management. For example, as mentioned above, a directive transmitter and receiver must dynamically align their beams toward each other. In today’s standards for both 5G and Wi-Fi, a serial sector sweep is used for initial beam alignment, to sequentially test different directions. This trial-and-error method becomes increasingly cumbersome as beams become narrower. In contrast, the aforementioned leaky-wave device can be used to rapidly track mobile clients by using the received spectral signature^64^ to estimate the receiver’s relative angle from the transmitter (see Fig. 6), a scheme which can be generalized to three-dimensional localization^74^. As another example, with arrays of sub-wavelength elements, one can envision a centimeter-scale surface with ~1000 independently controllable devices. This high oversampling yields new possibilities for dual-purposing communication and sensing: not only could one realize classical communication capabilities (e.g., beamforming and nulling of interferers or enhancing security), but one could also realize sensor functions (e.g., localization of users) with the same device^75^.

Likewise, beam steering must also incorporate cases in which a direct line-of-sight path is not available. As discussed above, an IRS could be used to realize a reflected path thereby increasing coverage and avoiding blockage. However, building the device is not enough; in order to function properly, the network’s control plane must discover and configure this path, including properties of the IRS itself: for example, if the IRS provides a non-specular reflection, it must know the targeted incoming and outgoing angles. Since a network may alternate serving users in time, the IRS would need to reconfigure not only due to user mobility, but also according to which users are transmitting and receiving. Beam steering devices must also consider the new wavefronts described in Section 3. For example, in typical demonstrations of beams such as OAM^39^, the transmitter, and receiver are manually aligned and are typically placed broadside. To employ such wavefronts in a mobile network will require adaptation not only for location, but also for the relative orientations of the transmitter and receiver when they are not ideally oriented.

As noted above, the aforementioned techniques based on the idea of an IRS have previously been considered for use at lower frequencies, but their implementation takes on new urgency at these higher frequencies. In addition, there are some approaches which have been more widely employed at lower frequencies, and which can also offer valuable capabilities in the THz range. One good illustration is the assessment of angle-of-arrival for a mobile user via cooperative estimation of spatial correlations among multiple antennas, for example in a massive MIMO architecture. This possibility has recently been considered by Peng et al.^76^ in the context of a THz network. Such legacy control-plane methods can play an important role, but will in general need some rethinking in view of the highly directional nature of transmissions in these networks, as well as the possibility (discussed above) that the user could be in the near field of the array.

Today’s wireless networks provide multi-user capabilities, in which an access point or base station transmits to (or receives from) multiple users simultaneously in order to increase aggregate data rate and decrease latency. Realizing this capability with THz frequencies will require two new advances. First, waveforms and modulation formats must be designed to support simultaneous transmission, incorporating that users will not be co-located and will be using directional transmissions. In some downlink cases, spatial separation of receivers combined with narrow beams may provide a simple starting point. Yet, for the uplink, and even for the downlink when users are close together, interference and co-stream management must be carefully controlled. Second, even if a network has the physical capability to realize a multi-user transmission, control plane mechanisms are needed to coordinate the transmission. Namely, the control plane must identify and localize the users, determine the appropriate spectrum and modulations to use, trigger the transmission at the correct time, and so on. In some cases, these functions are sufficiently similar to those of existing networks that comparable methods can be used; in other cases, entirely new protocols will need to be developed. For example, while traditionally medium access control protocols are driven by the transmitter, an alternative procedure based on receiver-initiated link synchronization, in which a receiver periodically polls potential transmitters as its antenna sweeps / scans the space, can increase the reliability and throughput and reduce latency^77^.

## Outlook

The many challenges discussed in this article have inspired a great deal of research over the last few years (only a small fraction of which could be included here). One challenge, not discussed above, involves the potential interference of wireless signals at these frequencies with existing (for the most part, passive) users involved in earth sensing or radio astronomy. Numerous research communities employ highly sensitive receivers to harvest information about the status of our atmosphere and the molecular composition of astronomical objects. It is critical that any active communication services that exploit frequencies above 100 GHz must be designed to avoid interference with these important existing communities^78^. Of course, because of the higher atmospheric attenuation and the high gain of transmitting antennas, issues of sharing and interference may be quite different at these high frequencies. More research is required, for example, to establish the limits for interference, or to demonstrate antenna configurations whose side lobes are designed to avoid interference with overhead satellites.

Unsurprisingly, the daunting nature of the technical challenges has also inspired some skepticism from some researchers in the field. A few have noted that R&D expenditures in THz systems from many of the major telecommunications companies remain only a small fraction of their total R&D budget. Of course, this is not surprising, since the market for these systems also remains tiny. At this juncture, one should not expect massive private sector investment in a technology that is probably at least a decade away. Another oft-stated concern relates to the need for such systems. Twenty years ago, a common refrain was that nobody would ever require frequency bands above 10 GHz for consumer applications; ten years ago, it was 60 GHz; today, with the emergence of the first commercial backhaul devices operating in D band^79^, the threshold has now moved to 140 GHz. In fact, we choose to regard this moving target as an optimistic indicator of the rapid progress in the field. This progress is embodied in exciting recent publications, including breakthrough new link demonstrations^80,81^ and rapid advances in solid-state device technology^82–85^.

Of course, there are valid reasons for concern; the challenges discussed in this article are indeed formidable. Nevertheless, we feel that the research results of the last few years have established that THz technologies are a promising foundation for future needs in wireless networks, which seem likely to exploit these frequencies for at least some of their key functions^86^. While many open questions remain, there is at this point a clear and compelling motivation to pursue the goal of THz wireless.

## References

[1] IEEE 802.11 Working Group. Enhancements for very high throughput for operation in license-exempt bands above 45 GHz. IEEE P802.11ay/D3.0 (2019).

[2] Kürner, T., Mittleman, D. M. & Nagatsuma, T. *THz Communications: Paving the Way towards Wireless Tbps* (Springer, 2021).

[3] Shafie, A. et al. Terahertz communications for 6G and beyond wireless networks: challenges, key advancements, and opportunities. *IEEE Network* (IEEE, 2022).

[4] Hirata A (2012). 120-GHz-band wireless link technologies for outdoor 10-Gbit/s data transmission. IEEE Trans. Microw. Theory Tech..

[5] Petrov V, Kürner T, Hosako I (2020). First standardization efforts for sub-terahertz band communications towards 6G. IEEE Commun. Mag..

[6] Balanis, C. A. *Antenna Theory: Analysis and Design* 4th ed. (John Wiley & Sons, 2016).

[7] Kleine-Ostmann T, Nagatsuma T (2011). A review on terahertz communications research. J. Infrared Millim. Terahertz Waves.

[8] Fu Z, Chen RT (1998). Five-bit substrate guided wave true-time delay module working up to 2.4 Thz with a packing density of 2.5 lines/cm2 for phased array antenna applications. Opt. Eng..

[9] International Telecommunication Union, Radiocommunication Sector (ITU-R), Recommendation P.676-11, Attenuation by atmospheric gases. https://www.itu.int/rec/R-REC-P.676-11-201609-I (2016).

[10] O’Hara JF, Grischkowsky DR (2018). Comment on the veracity of the ITU-R recommendation for atmospheric attenuation at terahertz frequencies. IEEE Trans. Terahertz Sci. Technol..

[11] Yang Y, Mandehgar M, Grischkowsky D (2015). THz-TDS characterization of the digital communication channels of the atmosphere and the enabled applications. J. Infrared Millim. Terahertz Waves.

[12] Fang Z, Hornbuckle M, Mittleman DM (2022). Secure communication channels using atmosphere-limited line-of-sight terahertz links. IEEE Trans. Terahertz Sci. Technol..

[13] Kallfass I (2015). 64 Gbit/s Transmission over 850 m Fixed Wireless Link at 240 GHz Carrier Frequency. J. Infrared Millim. Terahertz Waves.

[14] Wu, Q. et al. A 21 km 5 Gbps real time wireless communication system at 0.14 THz. In *42nd International Conference on Infrared, Millimeter, and Terahertz Waves (IRMMW-THz)* (IEEE, 2017).

[15] Sen P, Siles JV, Thawdar N, Jornet JM (2022). Multi-kilometer multi-gigabit-per-second (sub) terahertz communications. Nat. Electron..

[16] Federici JF, Ma J, Moeller L (2016). Review of weather impact on outdoor terahertz wireless communication links. Nano Commun. Netw..

[17] Sen, P. et al. Terahertz communications can work in rain and snow: Impact of adverse weather conditions on channels at 140 GHz. In *6th ACM Workshop on Millimeter-Wave and Terahertz Networks and Sensing Systems* 13–18 (ACM, 2022).

[18] Su K, Moeller L, Barat RB, Federici JF (2012). Experimental comparison of performance degradation from terahertz and infrared wireless links in fog. J. Opt. Soc. Am. A.

[19] Han C (2022). Terahertz wireless channels: a holistic survey on measurement, modeling, and analysis. IEEE Commun. Surv. Tutor..

[20] De Beelde B (2021). Material characterization and radio channel modeling at D-band frequencies. IEEE Access.

[21] Priebe S, Kannicht M, Jacob M, Kürner T (2013). Ultra broadband indoor channel measurements and calibrated ray tracing propagation modeling at THz frequencies. J. Commun. Netw..

[22] Abbasi NA (2022). THz band channel measurements and statistical modeling for urban D2D environments. IEEE Trans. Wirel. Commun..

[23] Sheikh, F., El-Hadidy, M. & Kaiser, T. Terahertz band: Indoor ray-tracing channel model considering atmospheric attenuation. In *IEEE International Symposium on Antennas and Propagation & USNC/URSI National Radio Science Meeting* 1782-1783 (IEEE, 2015).

[24] Ma J, Shrestha R, Moeller L, Mittleman DM (2018). Channel performance of indoor and outdoor terahertz wireless links. APL Photon.

[25] Jansen C (2011). Diffuse scattering from rough surfaces in THz communication channels. IEEE Trans. Terahertz Sci. Tech..

[26] Ma J, Shrestha R, Zhang W, Moeller L, Mittleman DM (2019). Terahertz wireless links using diffuse scattering from rough surfaces. IEEE Trans. Terahertz Sci. Tech..

[27] Messenger R, Strecker K, Ekin S, O’Hara JF (2021). Dispersion from diffuse reflectors and its effect on terahertz wireless communication performance. IEEE Trans. Terahertz Sci. Tech..

[28] Ma J (2018). Security and eavesdropping in terahertz wireless links. Nature.

[29] Mei Y, Ma Y, Ma J, Moeller L, Federici JF (2021). Eavesdropping risk evaluation on terahertz wireless channels in atmospheric turbulence. IEEE Access.

[30] Herold, C., Doeker, T., Eckhardt, J. M. & Kürner, T. Investigation of eavesdropping opportunities in a meeting room scenario for THz communications. In *16th European Conference on Antennas and Propagation (EuCAP)* (IEEE, 2022).

[31] Ju, S. & Rappaport, T. S. Sub-terahertz spatial statistical MIMO channel model for urban microcells at 142 GHz. In *IEEE Global Communications Conference* (*GLOBECOM*) (IEEE, 2021).

[32] Toskala, A., Holma, H. & Nakamura, T. *5G Technology: 3GPP New Radio* (John Wiley & Sons, 2020).

[33] Wu Q, Zhang S, Zheng B, You C, Zhang R (2021). Intelligent reflecting surface-aided wireless communications: a tutorial. IEEE Trans. Commun..

[34] Rouhi K, Hosseininejad SE, Abadal S, Khalily M, Tafazolli R (2021). Multi-channel near-field terahertz communications using reprogrammable graphene-based digital metasurface. J. Lightwave Technol..

[35] Zhang H (2022). Beam focusing for near-field multiuser MIMO communications. IEEE Trans. Wirel. Commun..

[36] Jo O, Kim J-J, Yoon J, Choi D, Hong W (2017). Exploitation of dual-polarization diversity for 5G millimeter-wave MIMO beamforming systems. IEEE Trans. Antennas Propag..

[37] Durnin J, Miceli JJ, Eberly JH (1988). Comparison of Bessel and Gaussian beams. Opt. Lett..

[38] Efremidis NK, Chen Z, Segev M, Christodoulides DN (2019). Airy beams and accelerating waves: an overview of recent advances. Optica.

[39] Zhou H (2022). Utilizing multiplexing of structured THz beams carrying orbital-angular-momentum for high-capacity communications. Opt. Express.

[40] Edfors O, Johansson AJ (2012). Is orbital angular momentum (OAM) based radio communication an unexploited area?. IEEE Trans. Antennas Propag..

[41] Li J-S, Chen J-Z (2021). Multi-beam and multi-mode orbital angular momentum by utilizing a single metasurface. Opt. Express.

[42] Khan MIW (2021). A 0.31-THz orbital-angular-momentum (OAM) wave transceiver in CMOS with bits-to-OAM mode mapping. IEEE J. Solid State Circuits.

[43] Venkatesh S, Lu X, Saeidi H, Sengupta K (2020). A high-speed programmable and scalable terahertz holographic metasurface based on tiled CMOS chips. Nat. Electron.

[44] Monroe, N. M. et al. Electronic THz pencil beam forming and 2D steering for high angular-resolution operation: a 98×98 unit, 265 GHz CMOS reflectarray with in-unit digital beam shaping and squint correction. In *IEEE International Solid-State Circuits Conference* (*ISSCC*) (2022).

[45] Shrestha R, Guerboukha H, Fang Z, Knightly E, Mittleman DM (2022). Jamming a terahertz wireless link. Nat. Commun..

[46] Woo, J. et al. Physical-layer security for THz communications via orbital angular momentum waves. In *IEEE Workshop on Signal Processing Systems* (*SiPS*) (IEEE, 2022).

[47] Hadani, R. et al. Orthogonal time frequency space (OTFS) modulation for millimeter-wave communications systems. In *International Microwave Symposium* (*IMS*) 681–683 (IEEE, 2017).

[48] Grischkowsky D, Keiding S, Exter MV, Fattinger C (1990). Far-infrared time-domain spectroscopy with terahertz beams of dielectrics and semiconductors. J. Opt. Soc. Am. B.

[49] Chen, P., Wang, Y. & Babakhani, A. A 4 ps amplitude reconfigurable impulse radiator with THz-TDS characterization method in 0.13 μm SiGe BiCMOS. In *IEEE MTT-S International Microwave Symposium* (*IMS*) (IEEE, 2016).

[50] Jornet JM, Akyildiz IF (2014). Femtosecond-long pulse-based modulation for terahertz band communication in nanonetworks. IEEE Trans. Commun..

[51] Bodet, D., Sen, P., Hossain, Z., Thawdar, N. & Jornet, J. M. Hierarchical bandwidth modulations for ultra-broadband communications in the terahertz band. *IEEE Trans. Wireless Commun*. (2022).

[52] Bosso, C. et al. Ultrabroadband spread spectrum techniques for secure dynamic spectrum sharing above 100 GHz between active and passive users. In *International Symposium on Dynamic Spectrum Access Networks* (*DySPAN*) 45–52 (IEEE, 2021).

[53] Abu-Surra, S. et al. End-to-end 6 G terahertz wireless platform with adaptive transmit and receive beamforming. in *IEEE International Conference on Communications Workshops* (*ICC Workshops*) 897–903 (IEEE, 2022).

[54] Watts CM (2014). Terahertz compressive imaging with metamaterial spatial light modulators. Nat. Photon.

[55] Sun, C., Chang, Q., Zhao, R., Wang, Y. & Wang, J. Terahertz imaging based on sparse MIMO array. In *International Conference on Microwave and Millimeter Wave Technology* (*ICMMT*) (IEEE, 2020).

[56] Singh, A., Andrello, M., Einarsson, E., Thawdar, N. & Jornet, J. M. Design and operation of a smart graphene–metal hybrid reflectarray at THz frequencies. In *14th European Conference on Antennas and Propagation* (*EuCAP*) (IEEE, 2020).

[57] Bandurin DA (2018). Resonant terahertz detection using graphene plasmons. Nat. Commun..

[58] Tu NH (2020). Active spatial control of terahertz plasmons in graphene. Commun. Mater..

[59] Jornet JM, Akyildiz IF (2013). Graphene-based plasmonic nano-antenna for terahertz band communication in nanonetworks. IEEE J. Sel. Areas Commun..

[60] Yeang, C.-P., Wornell, G. W. & Zheng, L. Oversampling transmit and receive antenna arrays. In *International Conference on Acoustics, Speech and Signal Processing* 2522–2525 (IEEE, 2010).

[61] Jackson DR, Caloz C, Itoh T (2012). Leaky wave antennas. Proc. IEEE.

[62] Ma J, Karl NJ, Bretin S, Ducournau G, Mittleman DM (2017). Frequency-division multiplexer and demultiplexer for terahertz wireless links. Nat. Commun..

[63] Matsumoto H, Watanabe I, Kasamatsu A, Monnai Y (2020). Integrated terahertz radar based on leaky-wave coherence tomography. Nat. Electron.

[64] Ghasempour Y, Shrestha R, Charous A, Knightly E, Mittleman DM (2020). Single-shot link discovery for terahertz wireless networks. Nat. Commun..

[65] Li R, Yan H, Cabric D (2022). Rainbow-link: beam-alignment-free and grant-free mmW multiple access using true-time-delay array. IEEE J. Sel. Areas Commun..

[66] Nguyen, H. V., Abielmona, S., Rennings, A. & Caloz, C. Pencil-beam full-space scanning 2D CRLH leaky-wave antenna array. In *International Symposium on Signals, Systems and Electronics* 139–142 (IEEE, 2007).

[67] Kamoda H, Iwasaki T, Tsumochi J, Kuki T, Hashimoto O (2011). 60-GHz electronically reconfigurable large reflectarray using single-bit phase shifters. IEEE Trans. Antennas Propag..

[68] Zhao Y (2019). High-speed efficient terahertz modulation based on tunable collective-individual state conversion within an active 3 nm two-dimensional electron gas metasurface. Nano Lett..

[69] Shaikhanov, Z., Hassan, F., Guerboukha, H., Mittleman, D. & Knightly, E. Metasurface-in-the-middle attack: from theory to experiment. In *15th ACM Conference on Security and Privacy in Wireless and Mobile Networks* (*WiSec ‘22*) 257–267 (ACM, 2022).

[70] Kanhere, O. & Rappaport, T. S. Outdoor sub-THz position location and tracking using field measurements at 142 GHz. In *IEEE International Conference on Communications* (*ICC*) (IEEE, 2021).

[71] Zhao, M. et al. Through-wall human mesh recovery using radio signals. In *IEEE/CVF International Conference on Computer Vision* (*ICCV*) 10112–10121 (IEEE, 2019).

[72] Lin, F. et al. Cardiac Scan: A non-contact and continuous heart-based user authentication system. In *23rd Annual International Conference on Mobile Computing and Networking* (*MobiCom ‘17*) 315–328 (ACM, 2017).

[73] Kanhere O, Rappaport TS (2021). Position location for futuristic cellular communications: 5G and beyond. IEEE Commun. Mag..

[74] Saeidi H, Venkatesh S, Lu X, Sengupta K (2021). THz prism: one shot simultaneous localization of multiple wireless nodes with leaky-wave THz antennas and transceivers in CMOS. IEEE J. Solid-State Circuits.

[75] Domae, B. W., Boljanovic, V., Li, R. & Cabric, D. Machine learning prediction for phase-less millimeter-wave beam tracking. In *IEEE 23rd International Workshop on Signal Processing Advances in Wireless Communication* (*SPAWC*) (IEEE, 2022). 10.1109/SPAWC51304.2022.9833935

[76] Peng B, Guan K, Kürner T (2018). Cooperative dynamic angle of arrival estimation considering space–time correlations for terahertz communications. IEEE Trans. Wirel. Commun..

[77] Xia Q, Hossain Z, Medley M, Jornet JM (2021). Link-layer synchronization and medium access control protocol for terahertz-band communication networks. IEEE Trans. Mob. Comput..

[78] Xing Y, Rappaport TS (2021). Terahertz wireless communications: co-sharing for terrestrial and satellite systems above 100 GHz. IEEE Commun. Lett..

[79] Singh, A. et al. A D-band radio-on-glass module for spectrally-efficient and low-cost wireless backhaul. In *IEEE Radio Frequency Integrated Circuits Symposium* (*RFIC*) 99–102 (IEEE, 2020).

[80] Kürner, T. & Kawanishi, T. Demonstrating 300 GHz wireless backhaul links – The ThoR approach. In *47th International Conference on Infrared, Millimeter and Terahertz Waves* (*IRMMW-THz*) (IEEE, 2022).

[81] Maes, D. et al. UTC photodiodes on silicon nitride enabling 100 Gbit/s Terahertz links at 300 GHz. In *European Conference on Optical Communication* (*ECOC*) (IEEE, 2022).

[82] Rodwell, M. et al. Transistors for 100-300 GHz wireless. In *IEEE 51st European Solid-State Device Research Conference* (*ESSDERC*) (IEEE, 2021).

[83] Wang, C. & Rebeiz, G. A 2-channel 136-156 GHz dual down-conversion I/Q receiver with 30 dB gain and 9.5 dB NF using CMOS 22 nm FDSOI. In *IEEE Radio Frequency Integrated Circuits Symposium* (*RFIC*) (IEEE, 2021).

[84] Chen Z, Choi W, O KK (2022). 300-GHz double-balanced up-converter using asymmetric MOS varactors in 65-nm CMOS. IEEE J. Solid-State Circuits.

[85] Mehta, Y., Thomas, S. & Babakhani, A. A 140–220-GHz low-noise amplifier with 6-dB minimum noise figure and 80-GHz bandwidth in 130-nm SiGe BiCMOS. *IEEE Microwave Wireless**Comp. Lett*. **33**, 200–203 (2022).

[86] Pang X (2022). Bridging the terahertz gap: photonics-assisted free-space communications from the submillimeter-wave to the mid-infrared. J. Lightwave Technol..

